# Climate change and inequality

**DOI:** 10.1038/s41390-024-03153-z

**Published:** 2024-06-24

**Authors:** Ella Sandrine Parsons, Ashley Jowell, Erika Veidis, Michele Barry, Sonoo Thadaney Israni

**Affiliations:** 1 Sean N. Parker Center for Allergy and Asthma Research at Stanford University, Stanford, CA USA; 2 Stanford University School of Medicine, Stanford, CA USA; 3 Center for Innovation in Global Health, Stanford University, Stanford, CA USA; 4 PRESENCE Canter, Stanford University School of Medicine, Stanford, CA USA

## Abstract

**Abstract:**

This review explores how climate change is manifesting along existing lines of inequality and thus further exacerbating current health disparities with a particular focus on children and future generations. Climate change risk and vulnerability are not equally distributed, nor is the adaptive capacity to respond to its adverse effects, which include health consequences, economic impacts, and displacement. Existing lines of inequality are already magnifying the adverse effects of climate change. Today’s children and future generations will experience a disproportionate number of adverse climate events than prior generations, especially children in lower-income populations, communities of color, and Indigenous communities. In order to mitigate the crisis of inequity accompanying the climate crisis, systemic action must be taken on a global scale – with a focus on protecting children and future generations, and in empowering youth-led environmental activism and engagement in climate policy.

**Impact statement:**

- Our review offers a current summary of the ways in which inequality is manifesting with respect to climate change in children and future generations.
- Rather than use a systematic review, we opted to use a theoretical framework to guide our review. We divided the effects of climate change into three effect pathways: via disruptions in (i) climate and weather, (ii) ecosystems, and (iii) society.
- By dividing our review in this theoretical framework, we can better suggest targeted public health interventions at each effect level. Furthermore, we are able to successfully identify literature gaps and areas of future research.

## Introduction

The adverse effects of human driven climate change are distributed unequally and places a disproportionate burden on already vulnerable populations. Women, lower income populations, and BIPOC (Black, Indigenous, and People of Color) communities have some of the highest risk of adverse effects associated with climate change.^1^ Children and future generations experience a disproportionate burden of climate change compared to prior generations. Regional climates, local geography, and existing inequalities of colonialism are some of many factors that will compound vulnerability to climate change.

While certain populations may encounter marginal benefits – such as longer growing seasons and milder weather – populations across the globe are increasingly threatened by extreme weather, drought, rising sea levels, extreme heat, food insecurity, resource scarcity, and disease. Populations particularly at risk include those in certain island states, people living in the Global South, including the African continent and South Asia region, and Indigenous populations.^2^ Not only are climate impacts inequitably distributed; populations’ adaptive capacities differ as well. For the purposes of this review, we will define adaptive capacity as the ability to adapt to climate change, through infrastructure, public health interventions, and individual adaptations. Existing inequalities and structural determinants will dictate who has financial access to innovative climate adaptations. Access to adaptive technologies to combat the adverse effects of climate change are essential to protect children, as children lack the necessary autonomy and capacity to protect themselves and instead are reliant on their families, communities and state resources. Moreover, children are also uniquely vulnerable to many of the health threats presented by climate change including pollution, malnutrition, susceptibility to disease, and mental health threats – as highlighted below. This is especially the case for children living in settings with other existing threats to health; children without access to clean air, clean water, and adequate nutrition and children already contending with a high burden of disease are more likely to be vulnerable to new climate-related threats.

**Fig. 1:**
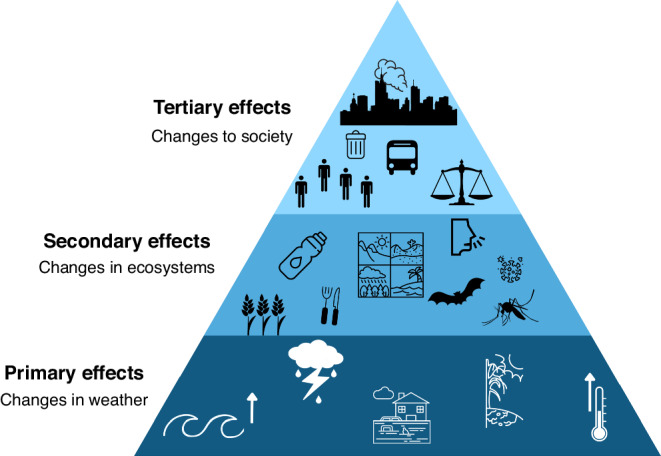
Levels of climate change effects. Climate change is a multifaceted issue that will impact people through a variety of mechanisms. To better understand the various ways that climate change will impact pediatric populations we identified a three-ringed pyramid of effects. The bottom tier includes the primary effects of climate change relating to the direct weather and climate changes that will occur because of human induced climate change. Primary effects include but are not limited to rising sea levels, extreme weather events, rising temperatures, and drought. The secondary effects, shown on the second rung will be changes dependant on the primary effects of climate change and will result in in changes in ecosystems, including but limited to disruptions in food and water systems and altering disease transmission routes through altered ecologies. In the highest level we discuss the tertiary effects and long-term social changes, arising through changes in the climate and ecosystems. Tertiary effects will arise as a result of complex, socio-political, economic and cultural responses as a result of climate change. This framework is conceptually related to the “primary”, “secondary” and “tertiary” health effect framework, upon which most of this book is based.

In this review, we highlight how climate change will continue to impact children’s wellbeing through a variety of pathways.^3^ We propose a conceptual framework with three effect pathways: via disruptions in (i) weather and climate, (ii) ecosystems, and (iii) society (Fig. 1). As inequality can result in illness, health inequities and premature deaths, we must prioritize how inequality will manifest with respect to varying levels of climate change vulnerabilities and a population’s capacity to cope, respond, and adapt to climate change to protect future generations. Not only will the adverse effects of climate change impact populations differently, but climate change will exacerbate historical and structural inequality, unless systematic action is taken across the globe to protect future generations (Table 1).

**Table 1:** Paper Overview.

Sections	Sub sections	Contents
1 Introduction	1.1 Primary Effects from Weather and Climate	• Define primary effects
	1.2 Secondary Effects from Ecosystems Disruptions	• Define secondary effects
	1.3 Tertiary Effects: Disruptions in Societies	• Define tertiary effects
2. Health Impacts	2.1 Health Effects of Altered Water Access	• Water scarcity • Water contamination • Waterborne disease burden in children
	2.2 Food Systems and Nutrition	• Effects of malnutrition in children • Distribution of food system disruption
	2.3 Effects of Wildfires on Health	• Health impacts of wildfire smoke in children • Risk factors of wildfire smoke
	2.4 Altered Disease Distribution via Ecosystem Disruptions	• Allergic Disorders • Zoonotic Diseases • Vector-Borne Diseases
	2.5 Mental Health Effects of Climate Change	• Depression • Anxiety • PTSD
	2.5 Social Determinants of Health and Climate Refugees	• Displacement and climate refugees • Migrant health disparities • Social Determinants of Health
3.0 Adaptive Capacity	3.1 Primary Adaptations to Extreme Weather Events	• State solutions to extreme weather • Global Inequality • Family level adaptations • Risk and unhoused families
	3.2 Adaptations to Health Impacts of Climate Change	• Public Health Interventions • Sanitation • Vector Management
	3.3 Call for Policy Changes in Response to Climate Change	• Youth Climate Movement • Policy Recommendations
Conclusion		

This table displays the overview of the review. The review is broken into three parts, an introduction, which includes an overview of the primary, secondary and tertiary effects associated with climate change, the respective health impacts associated with climate change and the respective adaptive capacities to climate change. Each of these three sections will explore the respective effects in pediatric populations and examine how inequality will exacerbate the adverse effects of climate change.

### Primary effects from weather and climate

The Intergovernmental Panel on Climate Change (IPCC) predicts that the global average temperature will increase between 1.5 and 5.7°C by 2100, due to anthropogenic greenhouse gas emissions.^1^ Children will continue to face extreme heat, drought, heavy precipitation, and other extreme weather events in greater frequency than prior generations as a result of human-induced climate change. The African continent is as likely to experience the most extreme effects of droughts compared to other continents.^4^ Extreme heat is particularly dangerous for children, who have a higher percent body water than adults, and who also take longer to produce sweat, in turn making them susceptible to heat stroke and dehydration.^5^ Heat-related mortality has increased across all regions worldwide.^6,7^ Due to increases in heavy precipitation and rising ocean levels, there has been an increase in damage from severe coastal storms.^6^ Global sea levels have risen about 90 mm in the past 25 years; the rate of this rise is steadily increasing.^8^ Rising sea levels and associated flooding, along with extreme precipitation events in dense urban areas is expected to displace millions of people in coming decades.^9^ According to a study by the World Bank, 2.2 billion people are already exposed to some level of flood risk.^10^ This risk is not distributed equally across the globe; 965 million people in Asia and the Pacific will experience high flooding risks.^10^ Furthermore, individuals in regions with high flood vulnerability are 15 times more likely to experience mortality than regions of low flood vulnerability.^6,10^

### Secondary effects from ecosystems disruptions

Changes in weather due to human-induced climate change will result in long-term ecosystem disruption. Increases in heat and drought will harm food and water systems and agriculture more broadly, impacting children’s access to necessary nutrition for healthy development.^1^ Higher temperatures and drought will increase the frequency, timing and location of wildfires and their toxic smoke, which we will explore further in this section.^11^ Changed ecosystems will alter disease distributions, changing and increasing the prevalence of asthma and allergies, vector-borne illness, and zoonotic diseases as well. Increased disease frequency can directly impact children, effect their quality of life, increase the risk of long-term disability and exacerbate existing health and other inequalities globally.

### Tertiary effects: disruptions in societies

The effects of climate change on society are somewhat nebulous to understand and must be contextualized within existing geopolitical trends. Understanding the ways in which climate change will alter society is essential to understanding the long-term well-being of future generations. Stable, families, schools, neighborhoods and local communities are essential in fostering healthy development of children.^12^ Geopolitical stability could falter in the future as a result of climate change, with potential increases in violent conflict, looming climate refugee crises, and decreases in biologic diversity.^12^ While technologic advances promise potential solutions to climate change, these adaptations will not equally benefit everyone. Technocentric approaches are frequently controlled by an elite and privileged group of experts, focusing on applying technologic solutions.^13^ However, technological solutions are almost always implemented unequally. Innovators must consider who these adaptive technologies benefit, who has the power and privilege, and who the decision makers are.

## Health impacts from climate change

### Health effects of altered water access

Access to water can be impacted by extreme heat, drought, and flooding. Historically, the African, Asian, and South American continents already have unequal access to clean drinking water compared to Europe and North America.^14^ Drought and water scarcity results in individuals lacking access to clean drinking water; instead relying on alternate sources of water, either bottled or unclean.^15^ Affluent populations can afford to purchase water from alternate sources or invest in expensive technologies like desalination.^14^ Flooding can also compromise access to clean drinking water through damaging water and sanitation systems, and increasing standing water.^16^ Flooding not only leads to compounding housing insecurity or damage, but can also contribute to water borne disease transmission, skin and soft tissue infections, and vector-borne disease spread. Globally, water-related diseases account for three million deaths annually, the majority of these deaths can be attributed to children between the ages of three and five.^15^ These frequent infections can also result in undernutrition.^15^

### Food systems and nutrition

Malnutrition is projected to worsen with climate change and children are vulnerable to these changes through water borne diseases (previously mentioned), food borne illnesses, and disruptions in agricultural systems.^1^ Currently, at least one third of children under five are undernourished or overweight; in addition, one half of children suffer from hidden hunger. As a result, millions of children globally are unable to grow and develop to their maximum potential.^17^ Food-borne illnesses are expected to increase as a result of climate change.^18^ Additionally, agricultural systems have already been impacted by changes in weather patterns.^6^ Parts of Latin America and the African continent have experienced an increase in acute food insecurity due to flooding and droughts.^6^ Higher levels of carbon dioxide have been associated with lower protein and nutrition in barley, wheat, rice, potatoes, soybeans and other foods.^19^ According to UNICEF’s reports, malnutrition can have devastating long-term and permanent effects on children, including but not limited to, stunting, wasting, hidden hunger, anemia, diabetes, obesity, depression, and even death.^17^ Unfortunately, children and young people face the greatest burden particularly in impoverished and marginalized communities, which results in vicious cycles of poverty and malnutrition for future generations.^17^

### Effects of wildfires on health

Children are at high risk for the adverse effects of wildfires. Drought, global increases in temperature, lightning and wind speed, and changes in precipitation have facilitated an increase in wildfires globally.^11^ Wildfire smoke is more dangerous than normal particulate matter as it contains more pro-inflammatory molecules and oxidative components. Children are more susceptible to smoke because children spend more time outdoors, they have immature immune and respiratory systems, and higher breathing rate relative to body size.^11^ Through the inhalation of fine particulate matter (PM_2.5_), children’s developing lungs can experience greater oxidative stress and greater inflammation, which in turn could increase a child’s risk for asthma, lung, or cardiovascular damage.^20,21^ Furthermore, wildfire smoke has been associated with increases in hospitalizations due to pulmonary disease, upper respiratory infection, and increased risk for both low birth weight and pre-term birth in the general public.^11^ Additionally, children are particularly vulnerable to the long term psychological consequences of wildfires.^11^

Wildfire smoke does not affect everyone equally. People in low-income areas are at greater risk for wildfires, meaning a child’s vulnerability to wildfires will likely be dictated by their parents and caregivers socio-economic status.^11^ The mechanism between poverty and wildfire smoke is still poorly understood; disproportionate effects in low-income neighborhoods could be explained by the lack of highly efficient air filters, poorly ventilated homes or schools, stress, pre-existing structural determinants of health or a combination of any or all of these factors.^11,22^ Wildfire smoke is just one pathway in which children can be exposed to dangerous levels of PM_2.5_; children can experience similar health effects via ambient air pollution exposure.

### Altered disease distribution via ecosystem disruptions

#### Allergic disorders

The geographical distribution of infectious zoonotic and vector borne diseases is changing due to human induced climate change. An increase in chronic conditions such as asthma and allergy has already been attributed to climate change.^23^ Rising temperatures generally mean longer growing seasons, faster plant growth, earlier pollen seasons and therefore an increase in pollen quantity and allergenicity, which could in turn result in increased severity of allergy symptoms and prevalence of allergic disorders; therefore, more children risk growing up with atopic disorders.^23^ Furthermore, thunderstorms can illicit increased levels of mold spores, which can trigger asthma in many individuals.^23^

#### Zoonotic diseases

**Fig. 2:**
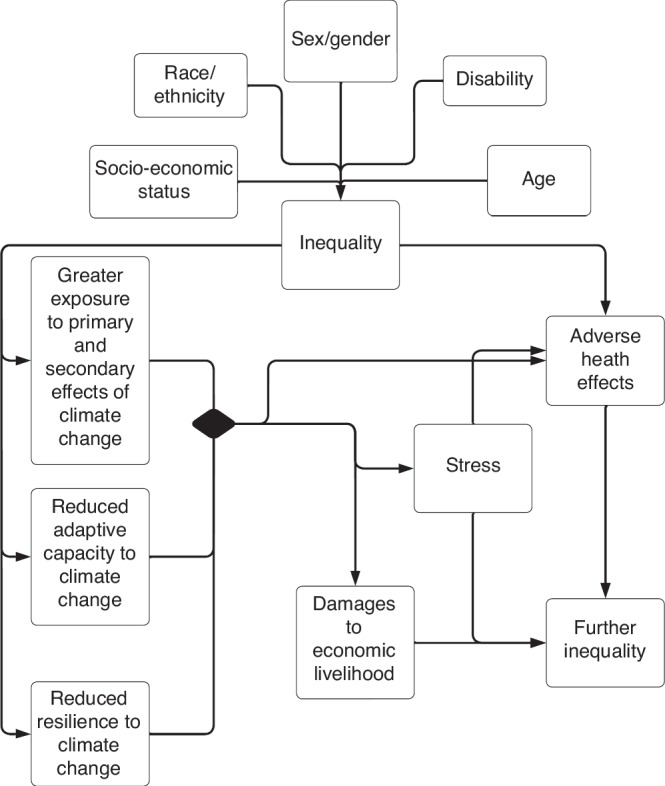
Mechanisms of vulnerability for vector-borne and other diseases. This figure shows a directed acyclic graph, or a causal diagram outlining a possible mechanism in which, changes in climate can result in altered land use, changes in favorable conditions for vectors can result in an increase in zoonotic and vector borne diseases. Furthermore, this figure illustrates how a family’s livelihood and economic status can predispose a child to risk for zoonotic and vector borne disease risks.

Zoonotic diseases are likely increasing due to climate change.^24^ Extreme weather, altered land use, intensive farming and population changes all increase the risk of spillover events.^25^ Spillover events often occur when people are in close contact with wildlife or livestock; sometimes in pursuit of bushmeat (Fig. 2).^25^ Zoonotic diseases (including SARS-CoV-2) disproportionately destroy vulnerable communities. One example: migrant children were excluded from COVID-19 vaccine rollout in many European Union (EU) member state rollout programs.^26^ Unless changes are made to our global health infrastructure, children’s well-being during future outbreaks will similarly be determined by existing structural inequalities.

#### Vector-borne diseases

Children are particularly vulnerable to vector-borne diseases due to the increased amount of time children spend outdoors.^27^ Climate change has already resulted in increases in vector borne diseases across numerous vectors.^25,28^ Warmer temperatures facilitate development and mortality rates of many mosquito vectors.^29^ However, relationship between precipitation and vector borne illnesses is more convoluted and varies with respect to socio-ecological landscapes.^29^ Increases in rainfall can increase *Aedes aegypti* breeding locations, as these disease carrying mosquitoes prefer to breed in proximity to human settlements in containers that hold water, such as trash, water basins, or puddles.^29^ Increases in *Ae. Aegypti mosquitoes* can result in increases in arboviruses.^29^ Conversely, drought can also creating more breeding loci for mosquitoes when people could store water in containers around the home.^29^ Furthermore, malaria has increased in African and South American continents and dengue has increased in the Asian continent and Brazil due to climate change (Fig. 2).^28^ Malaria alone accounts for roughly one million deaths annually primarily in pregnant women and children.^27^ Additionally, African sleeping sickness and Leishmaniasis have expanded to new regions where these diseases had previously never been found.^28^ In the United States, the frequency and distribution of tick-borne illnesses has increased.^18^ Tick-borne illnesses such as lyme disease, can have numerous long term consequences especially if undetected; the relationship between climate change and Lyme disease is complicated and is expected to increase in certain regions of the United States.^30^ These changes in vector diseases can be potentially catastrophic to vulnerable populations. For example, Rift Valley Fever Virus has increased due to changes in rainfall and has resulted in people losing their livelihood to the virus (Fig. 2).

### Mental health effects of climate change

Studies have found that across nations, climate anxiety and dissatisfaction with government responses are widespread in young people.^31^ Eco-anxiety, despair, hopelessness, guilt and sadness can impede children’s ability to function well.^31–33^ Children are uniquely vulnerable to the mental health effects of climate change. Depression, anxiety, and PTSD are most commonly associated with climate-related events.^33^ Climate change can impact mental health through both direct and indirect pathways.^33^ For example, acute severe weather events or longer lasting climate events can cause disruptions to daily life, including but not limited to unemployment, poverty, and family separation.^33^ Children and adolescents, particularly those in LMICs, are particularly vulnerable to the mental health impacts of climate change compared to children in high income countries (HIC).^33^ Children experience mental health disorders differently than adults; for example, children are likely to exhibit more severe symptoms than adults.^32^ Differences in PTSD symptoms could be due to either biological differences or reduced resilience or comprehensive understanding of the nuanced threats of climate change.^32^

### Structural determinants of health and climate refugees

One hundred and fifty million people are expected to be displaced by climate change by 2050, seeking refuge in regions and foreign countries perceived as more favorable.^34^ Millions of children around the world are on the move currently as a result of slow-onset disasters, environmental degradation and sudden-onset disasters exacerbated by climate change.^26^ Most of climate-change related displacement is internal; in 2020, 9.8 million weather-related internal displacements of children occurred.^26^ Mobility is a coping strategy to avoid the adverse effects of climate change.^26^

While young people in particular can benefit from migration when it provides the necessary opportunities to pursue aspirations and acquire new skillsets and education, children in high risk regions have limited options to migrate safely and legally across borders.^26^ Migrants are often blamed for increasing urban poverty, as they account for a large portion of the urban poor and often struggle to find adequate housing, employment, resources and governmental services.^26,35,36^ Displaced children often face barriers to attending school, accessing healthcare, child protection and other services that help build their resilience.^26^ Migrant children are actively denied access to necessary health care.^26^ For example, six EU member states actively restrict migrant children’s access to emergency care; only eight EU states grant all undocumented migrant children the same level of health care as their own citizens.^26^

Following migration and displacement, children may be met with cruel xenophobia and racism. For example, in the United States, all non-white children between the ages of 1–17 are experiencing increasing levels of racism, particularly Black and Indigenous children.^37^ Increases in racism are often associated with adverse long-term health outcomes.^37^ Furthermore, these adverse social experiences could further contribute to negative mental health impacts on refugees. Refugee children, particularly unaccompanied minors, are at high risk for PTSD.^38^

In a climate refugee crisis, rapid influxes of humans seeking safety will result in potentially rapid city expansion and unplanned urban sprawl. Poor city planning and massive waves of refugees have historically resulted in the creation of urban sprawl and large informal settlements. Urban sprawl can exacerbate climate change since longer commute distances translate to higher levels of emissions.^39^ Historically, as cities are industrialized, informal housing grows rapidly, but infrastructure such as water purification and sanitation lag, and green infrastructure, such as public transit are often added last.^40^

## Adaptive capacity

### Primary adaptations to extreme weather events

**Fig. 3:**
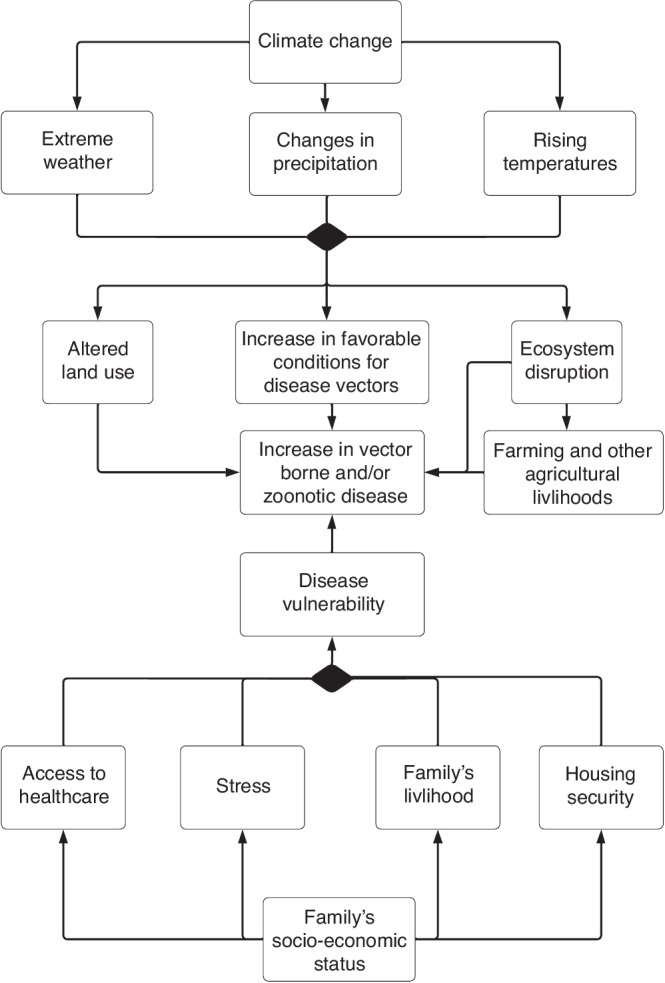
Proposed mechanism of furthering inequality. This figure represents a directed acyclic graph, or a causal diagram outlining a possible mechanism in which disadvantaged people are at higher risk from climate change. We highlight how inequality can manifest along numerous axes and identities. Inequality can result in greater exposure to the adverse effects of climate change, decreased resilience and adaptive capacity, which in turn can result in damages to livelihood, greater stress, and increased exposure to adverse health effects. The combined effect of losses to family livelihood, adverse health effects and stress, will likely result in only further inequality.

Equitable structural systems can help greatly to protect against climate extremes and provide physical barriers against the primary effects of climate change. At a state level, robust storm drains systems and flood walls can help prevent flooding and water contamination. At an individual level, heat waves can be easily endured inside well insulated and air-conditioned homes. Children in low and middle income countries make up roughly 85% of the world’s children and are at higher risk for the adverse effects of climate change, as their countries might lack the resources necessary to adapt to climate extremes.^3^ For example, fifty-nine percent of the population in the Netherlands lives 5 m below sea level,^41^ and despite this apparent vulnerability, the Dutch have the financial and engineering capacity to protect themselves and future generations from flooding in low-lying coastal areas, through costly infrastructure like their sea wall.^42^ Bangladesh, which has similar low-lying coastal regions, when faced with rising sea levels, lacks the capacity to construct costly sea walls. Unlike the Netherlands, which has relatively few people in housing insecurity, 47% of Bangladeshis live in informal settlements and 14% live in extreme poverty and are thus more likely to be displaced by climate change related events.^2,34^ Rising sea levels in Bangladesh could trigger a massive climate refugee crisis where 9% of the population - roughly 15 million people, the equivalent of 85% of the Dutch population- live 5 m below sea level.^41^ Physical barriers against extreme weather are the first line of defense for children facing the effects of climate change (Fig. 3).

While adaptive capacity varies greatly between countries, the primary effects of climate change will also vary within countries and communities along existing historically etched lines of inequality, deepened by centuries of colonialism and ungoverned capitalism. Even in an affluent United States, heat stroke occurs disproportionately in poorer neighborhoods that were racially segregated with State sanctioned and supported discriminatory housing policies, also known as redlining.^43^ Until the1968 Fair Housing Act, redlining denied BIPOC, poor and ethnic minorities from living in well-funded neighborhoods with discriminatory housing loans and racial covenants in housing deeds. The historic effects of redlining persist today in modern American cities.^43^ Studies have shown that poorer urban neighborhoods in historically redlined areas are hotter because they were inhumanely designed with less green space and greater population density.^43^ Affluent people may have estate like gardens, air conditioning and pools to luxuriate in; whereas, inhabitants of poorer neighborhoods might not even be able to afford adequate insulation or other cooling technologies.^43^ Children’s risk for heat stroke and dehydration directly hinges on their economic status and the neighborhoods they are born into.^43^ Well-constructed and maintained homes provide physical barriers against climate extremes. Those who lack access to well built housing and/or who were forcibly displaced are most at risk for adverse effects of climate change.^35,36,44^ Unhoused individuals and those living in informal settlements, particularly children, have little or no adaptive capacity. This ripples to lacking access to formal government infrastructure such as advanced warning systems, insurance for weather related damages, access to clean drinking water and waste management.^44^ Flooding in informal settlements can be disastrous due to inadequate drainage systems, destroying minimally habitable homes, lost personal assets, and access to clean drinking water.^44^

### Adaptations to health impacts of climate change

Climate change will continue to disrupt ecosystems with discriminatory water use, disrupted food access, wildfires, and cruel disease distributions. Preventing adverse health effects caused by unclean water, successful interventions must coordinate governmental and local efforts to focus on creating robust storm drainage systems, developing effective water sanitation practices, and ensuring equitable water access.^15^ To protect children against hunger and malnutrition, urgent strides must be made to protect our food systems. These include, but are not limited to, justice anchored governmental policies, equitable distributions and pricing of nutritious foods, regulation of food costs, and culturally conscious nutrition education programs. These essential interventions must be designed and assessed to guarantee equitable and resilient food systems and sufficient nutrition to children globally.^17^ Addressing the health effects of wildfire will require the collaboration of land and fire management, to create and assess clean air spaces for children such as houses and schools with adequate ventilation and air purification, indoor play spaces, availability of masks and air purifiers. Furthermore, granular level data, disaggregating by race/ethnicity, gender/sex, ability, zip codes and more is urgently needed to intelligently untangle the existing structural inequality in different sub-populations, the discriminatory effects of wildfires and PM_2.5_ on human and pediatric health. Ameliorating data collection will permit greater intentionality and accountability in solutions designed and delivered. Also, social programs for youth should prioritize JEDI (justice, equity, diversity and inclusion) when addressing issues such as self- esteem and self-efficacy. These should be central in a public health strategy where school curricula include historical successes of individuals from minority groups.^37^

Developing basic infrastructure is a necessary path to managing mosquito borne diseases. Vector prevention should ensure adequate drainage systems and roads that prevent water from collecting, and thus prevent mosquitoes from breeding.^45^ However, in the past century there has been a systematic divestment from improving central infrastructure in favor of technological investments in public health, such as vaccines and pharmaceuticals.^46^ There is not the same appeal for global health to invest in sewer systems as there is to develop profitable pharmaceuticals and vaccines. Unfortunately, many mosquito-borne diseases lack effective vaccines. Only yellow fever and Japanese encephalitis have moderately effective vaccines; malaria and dengue have vaccines which are mildly effective.^45,46^ Plus, there can be resistance to insecticides over time.^45,46^ Thus, children are likely to grow up in a world with more vector-borne diseases, insecticide resistance and limited effective vaccines. Policy, climate education, and mental health infrastructure are all necessary to protect the equitable mental health of children.^32,33^

### Call for policy changes in response to climate change

Children are our future and have the power to make a potential difference and curb the adverse effects of climate change for millenia. With climate anxieties rising, activism can serve as a means to overcome the hopelessness around climate change.^47^ Climate activism can take the form of strategic boycotts of certain products in favor of green alternatives, including but not limited to reducing automobile use, shifting to alternate fuel sources, or opting for greener lifestyles such as using reusable items or following a vegan diet.^47,48^ While an individual protest does not necessarily have a direct effect on climate change, these acts of civil disobedience can help pressure policy makers and corporations to take necessary action to reduce global warming.^48^ Public figures like Greta Thunberg have recentred discussions on climate change drawing attention to youth voices around climate change and inaction. One study found that people’s knowledge of Greta Thunberg’s activism translated to their likelihood to engage in climate activism.^49^ Millions of people have engaged in events lead by Thunberg, over seven million people participated in “Global Week for the Future”.^48^ Further research, data collection and analysis is needed to better understand the exact effects that activism has on climate policy and reducing long term emissions.

Policy holds a promise to curb many of the adverse effects of climate change, through meaningful interventions to promote public health, create physical barriers against the elements, and can reduce inequality through socio-political policies. Policies should:

- Encourage reducing emissions and incentivize switching to greener alternatives to slow the impact of climate change;
- Prioritize strong base infrastructure in which all individuals can benefit equitably
- Invest in structural barriers to battle extreme weather, such as robust storm drains to sea walls, or greening cities to reduce the effect of heatwaves
- Sustain and increase sanitation efforts globally, particularly in the global south to ensure clean drinking water for future generations
- Emphasize land management to promote equitable food systems adapted to changing climate extremrd
- Promote fuel and land management to reduce wild fires
- Require public policy and city planning to include the needs of populations displaced by climate change, to assure that adequate infrastructure is developed quickly to parallel housing needs;
- Expand healthcare access for all to include vulnerable children displaced by climate change.
- Allocate funding for medical research to develop lasting vaccines for diseases expected to increase or emerge, given climate change.

## Conclusion

Left unchecked, climate change will further exacerbate the existing climate inequalities - one where the privileged thrive with their lavish lifestyles and vulnerable communities are further decimated. Children are expected to face a disproportionate number of adverse climate events than adults. As the majority of the world’s children are in LMIC that lack the necessary adaptive capacity to protect its citizens, special attention, funding and policies must prioritize the needs of the world’s most vulnerable children and their health systems.^3^ We argue that action must be taken to protect vulnerable children and their communities, as they are facing greater losses to livelihood, displacement, and disproportionate disease burdens. To ensure equity for pediatric populations globally: equity, comprehensive tailored policies and funding strategies must be developed globally, including cost- and resource-sharing between HIC and LMIC and vulnerable communities. Furthermore, reparations are necessary to ensure equity, as HIC have disproportionately contributed to greenhouse gas emissions, but LMIC are more vulnerable to the adverse effects of climate change.^18,50^ HIC can use their political and economic power to assist in mitigating the adverse effects of climate change through lowering greenhouse gas emissions and facilitating the transition to clean energy. Unfortunately, foreign aid to assist with mitigating climate change is scarce, as promised funds from wealthy countries to partially compensate low-income countries for the damage from climate change have been repeatedly delayed since 2009.^51^

Sustained efforts must be prioritized to mitigate the adverse health impacts associated with climate change. Vaccines and protective technologies against vector borne and other climate-sensitive diseases are urgently needed to protect future generations. A multidisciplinary focus is required for granular data collection and reporting, research on the disease mechanisms of climate change and how it is magnified by poverty, which can empower front line stakeholders to inform and make decisions, redressing historical injustices as a priority, and accountability for needed change. We must continue to elevate the voices of young people as they will inherit the burden of climate change. Public health interventions must be prioritized and accessible to all, shifting away from investing primarily in costly technological solutions and instead to invest in infrastructure. Further research is needed to understand the effects of climate change at a more granular level of individuals, communities and societies. Vulnerable populations and young people must have the power to lead and direct efforts in research, policy, accountability, and decision making because they will bear the greatest burden of impending climate change.

“Never doubt that a small group of thoughtful, committed citizens can change the world; indeed, it’s the only thing that ever has.” – Margaret Meade
