# Correction: Climate change and inequality

**DOI:** 10.1038/s41390-024-03443-6

**Published:** 2024-07-29

**Authors:** Ella Sandrine Parsons, Ashley Jowell, Erika Veidis, Michele Barry, Sonoo Thadaney Israni

**Affiliations:** 1 Sean N. Parker Center for Allergy and Asthma Research at Stanford University, Stanford, CA USA; 2 Stanford University School of Medicine, Stanford, CA USA; 3 Center for Innovation in Global Health, Stanford University, Stanford, CA USA; 4 PRESENCE Canter, Stanford University School of Medicine, Stanford, CA USA

Correction to: *Pediatric Research* 10.1038/s41390-024-03153-z, published online 24 June 2024

The article “Climate change and inequality”, written by Ella Sandrine Parsons, Ashley Jowell, Erika Veidis, Michele Barry and Sonoo Thadaney Israni, was originally published electronically on the publisher’s internet portal on 24 June 2024 without open access. With the author(s)’ decision to opt for Open Choice the copyright of the article changed on 12 July 2024 to © The Author(s) 2024 and the article is forthwith distributed under a Creative Commons Attribution 4.0 International License, which permits use, sharing, adaptation, distribution and reproduction in any medium or format, as long as you give appropriate credit to the original author(s) and the source, provide a link to the Creative Commons licence, and indicate if changes were made

The images or other third party material in this article are included in the article’s Creative Commons licence, unless indicated otherwise in a credit line to the material. If material is not included in the article’s Creative Commons licence and your intended use is not permitted by statutory regulation or exceeds the permitted use, you will need to obtain permission directly from the copyright holder.

To view a copy of this licence, visit http://creativecommons.org/licenses/by/4.0/.

